# [1,2-Bis(diphenyl­phosphan­yl)ethane-κ^2^ *P*,*P*′]{2-[(4-nitro­benzoyl­meth­yl)diphenyl­phosphan­yl]phenyl-κ^2^ *C*,*C*′}palladium(II) trifluoro­methane­sulfonate–dichloro­methane–*n*-hexane (1/1/0.5)

**DOI:** 10.1107/S1600536811008075

**Published:** 2011-03-09

**Authors:** Corrado Rizzoli, Kazem Karami, Farzaneh Borzooie

**Affiliations:** a Dipartimento di Chimica Generale ed Inorganica, Chimica Analitica, Chimica Fisica, Universitá degli Studi di Parma, Viale G. P. Usberti 17/A, I-43124 Parma, Italy; b Department of Chemistry, Isfahan University of Technology, Isfahan 84156/83111, Iran

## Abstract

In the cation of the title compound, [Pd(C_26_H_19_NO_3_P)(C_26_H_24_P_2_)]CF_3_O_3_S·CH_2_Cl_2_·0.5C_6_H_14_, the Pd^II^ atom has a slightly tetra­hedrally distorted square-planar coordination geometry. The PdC_3_P and PdC_2_P_2_ five-membered metallacycles adopt envelope and twist conformations, respectively. In the crystal, inter­molecular C—H⋯O hydrogen bonds link cations and anions into a three-dimensional network. The dichloro­methane solvent mol­ecule is disordered over three orientations with a site-occupancy ratio of 0.5/0.3/0.2. The *n*-hexane solvent mol­ecule has a crystallographically imposed centre of symmetry.

## Related literature

For the synthesis and applications as catalysts of cyclo­palladated metal complexes, see: Rietling *et al.* (2002 ▶); Aguilar *et al.* (2008 ▶); Dupont *et al.* (2001 ▶); Chen *et al.* (2009 ▶). For *ortho*-palladated α-ketophospho­rus ylides complexes reported by our group, see: Karami *et al.* (2010 ▶); Karami, Rizzoli & Salah (2011 ▶); Karami, Rizzoli & Borzooie (2011 ▶). For related structures, see: Falvello *et al.* (1998 ▶, 1999 ▶); Shao *et al.* (1982 ▶).
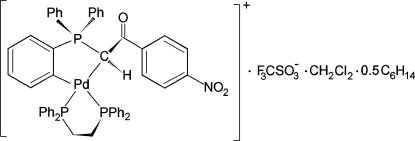


## Experimental

### 

#### Crystal data

[Pd(C_26_H_19_NO_3_P)(C_26_H_24_P_2_)]CF_3_O_3_S·CH_2_Cl_2_·0.5C_6_H_14_
*M* *_r_* = 1206.27
Monoclinic, 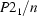

*a* = 12.4063 (7) Å
*b* = 14.2445 (8) Å
*c* = 31.3633 (17) Å
β = 91.6675 (9)°
*V* = 5540.2 (5) Å^3^
*Z* = 4
Mo *K*α radiation
μ = 0.62 mm^−1^
*T* = 294 K
0.19 × 0.16 × 0.10 mm

#### Data collection

Bruker APEXII CCD diffractometer
Absorption correction: multi-scan (*SADABS*; Bruker, 2008 ▶) *T*_min_ = 0.872, *T*_max_ = 0.955
62924 measured reflections
10529 independent reflections
8106 reflections with *I* > 2σ(*I*)
*R*_int_ = 0.036

#### Refinement

*R*[*F* ^2^ > 2σ(*F* ^2^)] = 0.029
*wR*(*F* ^2^) = 0.076
*S* = 1.04
10529 reflections
691 parameters
14 restraints
H-atom parameters constrained
Δρ_max_ = 0.37 e Å^−3^
Δρ_min_ = −0.38 e Å^−3^

### 

Data collection: *APEX2* (Bruker, 2008 ▶); cell refinement: *APEX2*; data reduction: *SAINT* (Bruker, 2008 ▶); program(s) used to solve structure: *SIR97* (Altomare *et al.*, 1999 ▶); program(s) used to refine structure: *SHELXL97* (Sheldrick, 2008 ▶); molecular graphics: *ORTEP-3 for Windows* (Farrugia, 1997 ▶) and *SCHAKAL97* (Keller, 1997 ▶); software used to prepare material for publication: *SHELXL97* and *PARST95* (Nardelli, 1995 ▶).

## Supplementary Material

Crystal structure: contains datablocks global, I. DOI: 10.1107/S1600536811008075/zj2004sup1.cif

Structure factors: contains datablocks I. DOI: 10.1107/S1600536811008075/zj2004Isup2.hkl

Additional supplementary materials:  crystallographic information; 3D view; checkCIF report

## Figures and Tables

**Table 1:** Hydrogen-bond geometry (Å, °)

*D*—H⋯*A*	*D*—H	H⋯*A*	*D*⋯*A*	*D*—H⋯*A*
C19—H19⋯O1^i^	0.93	2.54	3.284 (3)	138
C30—H30⋯O6^ii^	0.93	2.55	3.372 (4)	148
C39—H39*B*⋯O6^iii^	0.97	2.59	3.309 (4)	131

Symmetry codes: (i) 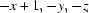; (ii) 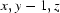; (iii) 
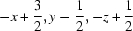
.

**Fig. 1.**
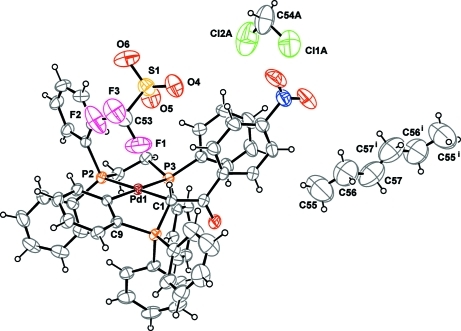
The asymmetric unit of the title compound, with displacement ellipsoids drawn at the 30% probability level. Only the major component of the disordered dichloromethane molecule is shown. Symmetry code: (i) -x, 1 - y, -z.

**Fig. 2.**
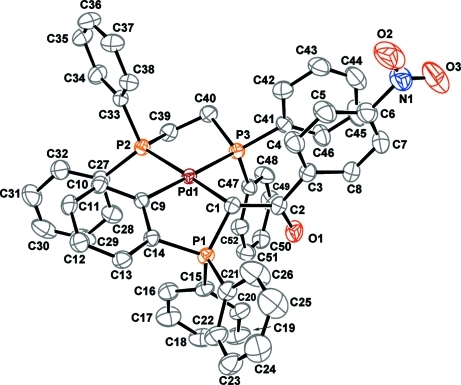
The cation of the title compound, showing the labelling scheme adopted. Displacement ellipsoids are drawn at the 30% probability level. Hydrogen atoms are omitted for clarity.

**Fig. 3.**
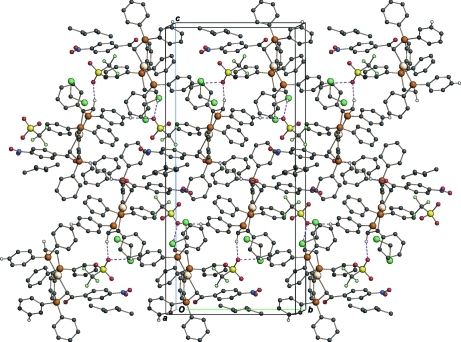
Crystal packing of the title compound approximately viewed along the a axis. Only the major component of the disordered dichloromethane molecule is shown. Hydrogen atoms not involved in intermolecular C—H···O hydrogen bonds (dashed lines) are omitted for clarity.

## supplementary crystallographic information

### Comment

The synthesis and characterization of cyclopalladated metal complexes (Rietling *et al.*, 2002) has attracted considerable attention due to their potential applications in organic synthesis and homogenous catalysis (Aguilar *et al.*, 2008; Dupont *et al.*, 2001; Chen *et al.*, 2009). As a continuation of our ongoing project devoted to the development of new catalysts based on *ortho*-palladated α-ketophosphorus ylides complexes (Karami *et al.*, 2010; Karami, Rizzoli & Salah, 2011; Karami, Rizzoli & Borzooie, 2011), we report herein the synthesis and crystal structure of the title compound.

The asymmetric unit of the title compound (Fig. 1) consists of one mononuclear complex cation, one trifluoromethanesulfonate anion, one disordered dichloromethane molecule and half an *n*-hexane lying on a centre of symmetry. In the cation (Fig. 2), the palladium(II) atom displays a slightly but not negligibly tetrahedrally distorted square planar coordination geometry, with atoms P2, P3, C1 and C9 displaced from the mean plane through the P_2_C_2_ core by -0.0061 (6), 0.0055 (6), -0.070 (2) and 0.069 (2) Å, respectively. The distortion from the regular square planar geometry is also indicated by the values of the *cis* and *trans* angles subtended at the metal, which range from 83.17 (8) to 100.49 (5)°, and from 175.31 (5) to 175.68 (5)°, respectively. The Pd–C bond lengths involving the aromatic and ylidic carbon atoms (Pd1–C9 = 2.0783 (19) Å; Pd–C1 = 2.1711 (19) Å) are in agreement with those observed in related cyclopalladated complexes (Falvello *et al.*, 1998; Falvello *et al.*, 1999; Karami *et al.*, 2010; Karami, Rizzoli & Salah, 2011; Karami, Rizzoli & Borzooie, 2011). The P1–C1 bond length (1.768 (2) Å) is significantly longer than that observed in the related free ylide (1.711 Å) of formula PPh_3_C(H)COPh (Shao *et al.*, 1982). The Pd1···P1 separation is 3.0241 (9) Å. The PdC_3_P five-membered metallacycle (Pd1/C9/C14/P1/C1) assumes an envelope conformation, with atom C1 displaced by 1.006 (2) Å from the mean planes of the remaining four atoms, whereas the PdC_2_P_2_ metallacycle (Pd1/P2/C39/C40/P3) adopts a twist conformation with the local twofold axis passing through the C39–C40 bond and the Pd atom. In the crystal structure (Fig. 3), cations and anions are linked into a three-dimensional network by intermolecular C—H···O hydrogen bonds (Table 1).

### Experimental

The title compound was obtained according to the procedure recently reported elsewhere (Karami, Rizzoli & Borzooie, 2011). Crystals suitable for X-ray analysis were obtained by slow evaporation of a dichloromethane/*n*-hexane (1:1 *v*/*v*) solution at room temperature.

### Refinement

All H atoms were placed in calculated positions and refined using a riding model, with C–H = 0.93–0.98 Å, and with *U*_iso_(H) = 1.2 *U*_eq_(C) or 1.5 *U*_eq_(C) for methyl H atoms. The dichloromethane solvent molecule was found to be disordered over three orientations (called *A*, *B*, and *C*) with site-occupancy factors of 1/2, 0.3 and 1/5, respectively. During the refinement, the C–Cl and Cl···Cl distances were constrained to 1.75 (1) and 2.75 (2) Å, respectively, and only the major component of disorder was refined anisotropically. The *n*-hexane molecule, which has crystallographically imposed centre of symmetry, showed rather high displacement ellipsoids, suggesting the presence of disorder. Attempts to model the molecule in terms of disordered contributors were unsuccessful, however. The molecule was therefore anisotropically refined by constraining the C–C bond lengths to 1.54 (1) Å, and the 1–3 C···C separations to 2.52 (2) Å.

### Crystal data

**Table:**

[Pd(C_26_H_19_NO_3_P)(C_26_H_24_P_2_)]CF_3_O_3_S·CH_2_Cl_2_·0.5C_6_H_14_	*F*(000) = 2468
*M**_r_* = 1206.27	*D*_x_ = 1.446 Mg m^−^^3^
Monoclinic, *P*2_1_/*n*	Mo *K*α radiation, λ = 0.71073 Å
Hall symbol: -P 2yn	Cell parameters from 1226 reflections
*a* = 12.4063 (7) Å	θ = 6.3–23.4°
*b* = 14.2445 (8) Å	µ = 0.62 mm^−^^1^
*c* = 31.3633 (17) Å	*T* = 294 K
β = 91.6675 (9)°	Irregular block, yellow
*V* = 5540.2 (5) Å^3^	0.19 × 0.16 × 0.10 mm
*Z* = 4	

### Data collection

**Table:**

Bruker APEXII CCD diffractometer	10529 independent reflections
Radiation source: fine-focus sealed tube	8106 reflections with *I* > 2σ(*I*)
graphite	*R*_int_ = 0.036
ω scans	θ_max_ = 25.7°, θ_min_ = 1.6°
Absorption correction: multi-scan (*SADABS*; Bruker, 2008)	*h* = −15→15
*T*_min_ = 0.872, *T*_max_ = 0.955	*k* = −17→17
62924 measured reflections	*l* = −38→38

### Refinement

**Table:**

Refinement on *F*^2^	Primary atom site location: structure-invariant direct methods
Least-squares matrix: full	Secondary atom site location: difference Fourier map
*R*[*F*^2^ > 2σ(*F*^2^)] = 0.029	Hydrogen site location: inferred from neighbouring sites
*wR*(*F*^2^) = 0.076	H-atom parameters constrained
*S* = 1.04	*w* = 1/[σ^2^(*F*_o_^2^) + (0.038*P*)^2^] where *P* = (*F*_o_^2^ + 2*F*_c_^2^)/3
10529 reflections	(Δ/σ)_max_ = 0.001
691 parameters	Δρ_max_ = 0.37 e Å^−^^3^
14 restraints	Δρ_min_ = −0.38 e Å^−^^3^

### Special details

**Table:**

Refinement. Refinement of *F*^2^ against ALL reflections. The weighted *R*-factor *wR* and goodness of fit *S* are based on *F*^2^, conventional *R*-factors *R* are based on *F*, with *F* set to zero for negative *F*^2^. The threshold expression of *F*^2^ > σ(*F*^2^) is used only for calculating *R*-factors(gt) *etc*. and is not relevant to the choice of reflections for refinement. *R*-factors based on *F*^2^ are statistically about twice as large as those based on *F*, and *R*- factors based on ALL data will be even larger.

### Fractional atomic coordinates and isotropic or equivalent isotropic displacement parameters (Å^2^)

**Table:**

	*x*	*y*	*z*	*U*_iso_*/*U*_eq_	Occ. (<1)
Pd1	0.756229 (11)	0.147230 (10)	0.131977 (5)	0.03814 (6)	
S1	0.90642 (7)	0.50943 (6)	0.15555 (3)	0.0883 (2)	
P1	0.81951 (4)	0.14269 (4)	0.039724 (17)	0.04289 (13)	
P3	0.57524 (4)	0.14972 (4)	0.152637 (18)	0.04526 (13)	
P2	0.78566 (4)	0.07474 (4)	0.196011 (17)	0.04514 (14)	
O1	0.57447 (13)	0.19304 (12)	0.03587 (6)	0.0709 (5)	
O2	0.5578 (3)	0.68752 (18)	0.07768 (10)	0.1340 (10)	
O3	0.4015 (3)	0.6445 (2)	0.06026 (13)	0.1685 (14)	
O4	0.8765 (2)	0.57210 (17)	0.12237 (9)	0.1226 (8)	
O5	0.82523 (18)	0.44856 (18)	0.17003 (8)	0.1183 (8)	
O6	0.9700 (3)	0.5548 (2)	0.18984 (9)	0.1499 (11)	
F1	0.9555 (2)	0.3873 (2)	0.09940 (9)	0.1675 (11)	
F2	1.0418 (2)	0.37281 (17)	0.16085 (10)	0.1651 (11)	
F3	1.08767 (16)	0.47955 (15)	0.11854 (7)	0.1293 (7)	
N1	0.4919 (3)	0.6307 (2)	0.06826 (9)	0.0947 (9)	
C1	0.74213 (16)	0.21915 (14)	0.07104 (6)	0.0428 (5)	
H1	0.7844	0.2770	0.0742	0.051*	
C2	0.63523 (17)	0.24779 (16)	0.05422 (7)	0.0487 (5)	
C3	0.60121 (18)	0.34836 (15)	0.05941 (7)	0.0524 (5)	
C4	0.6672 (2)	0.41801 (17)	0.07639 (9)	0.0697 (7)	
H4	0.7363	0.4030	0.0865	0.084*	
C5	0.6311 (3)	0.51037 (18)	0.07854 (10)	0.0794 (8)	
H5	0.6762	0.5574	0.0893	0.095*	
C6	0.5308 (3)	0.53044 (19)	0.06495 (9)	0.0731 (7)	
C7	0.4620 (2)	0.4644 (2)	0.04858 (9)	0.0828 (9)	
H7	0.3923	0.4807	0.0397	0.099*	
C8	0.4971 (2)	0.37316 (19)	0.04537 (8)	0.0694 (7)	
H8	0.4513	0.3276	0.0338	0.083*	
C9	0.91717 (15)	0.13543 (13)	0.11614 (6)	0.0398 (5)	
C10	1.00786 (17)	0.12915 (15)	0.14348 (7)	0.0502 (5)	
H10	0.9988	0.1295	0.1728	0.060*	
C11	1.11043 (17)	0.12245 (18)	0.12802 (8)	0.0608 (6)	
H11	1.1689	0.1186	0.1472	0.073*	
C12	1.12825 (18)	0.12141 (18)	0.08535 (8)	0.0641 (7)	
H12	1.1981	0.1167	0.0755	0.077*	
C13	1.04155 (18)	0.12737 (16)	0.05688 (7)	0.0545 (6)	
H13	1.0521	0.1268	0.0276	0.065*	
C14	0.93907 (16)	0.13423 (14)	0.07244 (6)	0.0424 (5)	
C15	0.76628 (17)	0.02642 (16)	0.03113 (6)	0.0490 (5)	
C16	0.8100 (2)	−0.04862 (17)	0.05335 (8)	0.0621 (6)	
H16	0.8659	−0.0395	0.0733	0.074*	
C17	0.7694 (3)	−0.13839 (18)	0.04549 (10)	0.0845 (9)	
H17	0.7980	−0.1894	0.0604	0.101*	
C18	0.6875 (3)	−0.1518 (2)	0.01586 (11)	0.0873 (10)	
H18	0.6610	−0.2120	0.0108	0.105*	
C19	0.6445 (2)	−0.0779 (2)	−0.00625 (9)	0.0789 (8)	
H19	0.5893	−0.0877	−0.0264	0.095*	
C20	0.68299 (19)	0.01173 (19)	0.00130 (7)	0.0618 (6)	
H20	0.6531	0.0623	−0.0136	0.074*	
C21	0.84920 (18)	0.18697 (18)	−0.01227 (7)	0.0545 (6)	
C22	0.8883 (2)	0.1285 (2)	−0.04356 (8)	0.0712 (7)	
H22	0.8942	0.0643	−0.0384	0.085*	
C23	0.9187 (2)	0.1647 (3)	−0.08236 (9)	0.0815 (9)	
H23	0.9486	0.1252	−0.1025	0.098*	
C24	0.9057 (3)	0.2541 (3)	−0.09099 (11)	0.1113 (12)	
H24	0.9286	0.2781	−0.1168	0.134*	
C25	0.8589 (3)	0.3115 (3)	−0.06240 (12)	0.1215 (13)	
H25	0.8428	0.3732	−0.0699	0.146*	
C26	0.8347 (3)	0.2789 (2)	−0.02164 (10)	0.1082 (12)	
H26	0.8090	0.3199	−0.0013	0.130*	
C27	0.85865 (19)	−0.03586 (15)	0.19613 (7)	0.0533 (6)	
C28	0.8165 (2)	−0.10762 (19)	0.17098 (9)	0.0715 (7)	
H28	0.7543	−0.0974	0.1544	0.086*	
C29	0.8663 (3)	−0.1941 (2)	0.17036 (11)	0.0857 (9)	
H29	0.8370	−0.2422	0.1537	0.103*	
C30	0.9574 (3)	−0.2089 (2)	0.19399 (11)	0.0962 (11)	
H30	0.9903	−0.2675	0.1937	0.115*	
C31	1.0012 (3)	−0.1393 (3)	0.21797 (11)	0.1123 (13)	
H31	1.0649	−0.1501	0.2336	0.135*	
C32	0.9523 (2)	−0.0517 (2)	0.21953 (9)	0.0830 (8)	
H32	0.9827	−0.0042	0.2363	0.100*	
C33	0.84562 (17)	0.15069 (15)	0.23661 (7)	0.0498 (5)	
C34	0.8534 (2)	0.1220 (2)	0.27935 (8)	0.0698 (7)	
H34	0.8303	0.0625	0.2871	0.084*	
C35	0.8953 (2)	0.1821 (3)	0.30950 (9)	0.0839 (9)	
H35	0.8999	0.1634	0.3379	0.101*	
C36	0.9302 (2)	0.2687 (3)	0.29844 (10)	0.0884 (9)	
H36	0.9601	0.3082	0.3192	0.106*	
C37	0.9221 (2)	0.2985 (2)	0.25752 (10)	0.0777 (8)	
H37	0.9457	0.3583	0.2504	0.093*	
C38	0.87898 (18)	0.24007 (17)	0.22645 (8)	0.0581 (6)	
H38	0.8723	0.2611	0.1984	0.070*	
C39	0.65511 (18)	0.04531 (17)	0.21851 (7)	0.0572 (6)	
H39A	0.6257	−0.0108	0.2050	0.069*	
H39B	0.6635	0.0338	0.2489	0.069*	
C40	0.58024 (18)	0.12801 (17)	0.21030 (7)	0.0556 (6)	
H40A	0.6071	0.1831	0.2254	0.067*	
H40B	0.5087	0.1138	0.2202	0.067*	
C41	0.48826 (17)	0.25194 (16)	0.14711 (7)	0.0513 (5)	
C42	0.5253 (2)	0.33604 (18)	0.16386 (9)	0.0713 (7)	
H42	0.5942	0.3398	0.1762	0.086*	
C43	0.4591 (3)	0.4154 (2)	0.16227 (9)	0.0848 (9)	
H43	0.4840	0.4720	0.1736	0.102*	
C44	0.3585 (3)	0.4099 (2)	0.14422 (11)	0.0940 (10)	
H44	0.3141	0.4625	0.1439	0.113*	
C45	0.3215 (2)	0.3273 (2)	0.12634 (12)	0.0943 (10)	
H45	0.2533	0.3243	0.1133	0.113*	
C46	0.38683 (19)	0.24915 (19)	0.12800 (9)	0.0733 (7)	
H46	0.3620	0.1932	0.1160	0.088*	
C47	0.49623 (16)	0.04970 (15)	0.13374 (7)	0.0484 (5)	
C48	0.40033 (18)	0.02404 (18)	0.15241 (9)	0.0669 (7)	
H48	0.3732	0.0610	0.1741	0.080*	
C49	0.3453 (2)	−0.05461 (19)	0.13946 (9)	0.0732 (7)	
H49	0.2799	−0.0693	0.1516	0.088*	
C50	0.3860 (2)	−0.11248 (18)	0.10840 (9)	0.0661 (7)	
H50	0.3497	−0.1669	0.1001	0.079*	
C51	0.4797 (2)	−0.08836 (18)	0.09037 (8)	0.0662 (7)	
H51	0.5077	−0.1269	0.0694	0.079*	
C52	0.53468 (18)	−0.00806 (16)	0.10225 (7)	0.0554 (6)	
H52	0.5983	0.0074	0.0889	0.067*	
C53	1.0025 (3)	0.4339 (3)	0.13228 (13)	0.1008 (10)	
C54A	0.3163 (9)	0.6781 (8)	0.2291 (3)	0.194 (5)	0.50
H54A	0.2741	0.7264	0.2427	0.232*	0.50
H54B	0.3922	0.6927	0.2334	0.232*	0.50
Cl1A	0.2831 (3)	0.6713 (2)	0.17539 (14)	0.1998 (15)	0.50
Cl2A	0.2875 (4)	0.5690 (5)	0.25025 (15)	0.288 (3)	0.50
C54B	0.2090 (16)	0.6089 (8)	0.2196 (4)	0.141 (6)*	0.30
H54C	0.2623	0.6138	0.1977	0.170*	0.30
H54D	0.1382	0.6202	0.2067	0.170*	0.30
Cl1B	0.2144 (9)	0.4956 (6)	0.2440 (3)	0.295 (5)*	0.30
Cl2B	0.2361 (6)	0.6898 (5)	0.2604 (2)	0.198 (2)*	0.30
C54C	0.2675 (17)	0.7357 (9)	0.2117 (4)	0.107 (6)*	0.20
H54E	0.3340	0.7619	0.2013	0.129*	0.20
H54F	0.2060	0.7657	0.1976	0.129*	0.20
Cl1C	0.2632 (13)	0.6113 (9)	0.2074 (5)	0.245 (6)*	0.20
Cl2C	0.2614 (9)	0.7397 (8)	0.2671 (3)	0.207 (4)*	0.20
C55	0.1874 (6)	0.3580 (5)	0.0169 (3)	0.281 (5)	
H55A	0.2628	0.3594	0.0248	0.421*	
H55B	0.1753	0.3134	−0.0057	0.421*	
H55C	0.1467	0.3400	0.0412	0.421*	
C56	0.1529 (5)	0.4517 (5)	0.0023 (2)	0.220 (3)	
H56A	0.1877	0.4664	−0.0241	0.265*	
H56B	0.1758	0.4980	0.0234	0.265*	
C57	0.0311 (5)	0.4582 (4)	−0.0047 (3)	0.221 (4)	
H57A	0.0165	0.4437	−0.0345	0.265*	
H57B	0.0003	0.4074	0.0116	0.265*	

### Atomic displacement parameters (Å^2^)

**Table:**

	*U*^11^	*U*^22^	*U*^33^	*U*^12^	*U*^13^	*U*^23^
Pd1	0.03380 (9)	0.04284 (10)	0.03775 (9)	−0.00153 (7)	0.00029 (6)	−0.00164 (7)
S1	0.0898 (6)	0.0880 (5)	0.0879 (6)	0.0087 (5)	0.0177 (5)	0.0037 (5)
P1	0.0401 (3)	0.0506 (3)	0.0378 (3)	−0.0059 (2)	0.0005 (2)	0.0001 (2)
P3	0.0354 (3)	0.0529 (3)	0.0476 (3)	−0.0014 (2)	0.0040 (2)	−0.0047 (3)
P2	0.0459 (3)	0.0482 (3)	0.0414 (3)	−0.0009 (2)	0.0010 (2)	0.0032 (2)
O1	0.0542 (10)	0.0624 (10)	0.0944 (13)	−0.0030 (8)	−0.0271 (9)	−0.0110 (10)
O2	0.193 (3)	0.0650 (16)	0.145 (2)	0.0284 (18)	0.019 (2)	0.0046 (16)
O3	0.173 (3)	0.112 (2)	0.220 (4)	0.076 (2)	−0.017 (3)	−0.022 (2)
O4	0.1203 (19)	0.1035 (18)	0.144 (2)	0.0192 (15)	0.0099 (16)	0.0336 (16)
O5	0.0866 (15)	0.135 (2)	0.135 (2)	−0.0087 (15)	0.0315 (14)	0.0224 (16)
O6	0.186 (3)	0.153 (2)	0.111 (2)	−0.027 (2)	0.0091 (19)	−0.0592 (18)
F1	0.154 (2)	0.170 (2)	0.178 (3)	−0.0203 (18)	0.0109 (19)	−0.098 (2)
F2	0.143 (2)	0.1191 (17)	0.236 (3)	0.0479 (15)	0.049 (2)	0.0631 (19)
F3	0.0921 (13)	0.1338 (17)	0.165 (2)	−0.0059 (12)	0.0491 (13)	0.0272 (15)
N1	0.120 (3)	0.079 (2)	0.0850 (19)	0.0314 (19)	0.0008 (18)	0.0077 (16)
C1	0.0426 (11)	0.0408 (11)	0.0448 (12)	−0.0048 (9)	−0.0013 (9)	0.0020 (9)
C2	0.0447 (12)	0.0530 (13)	0.0482 (12)	−0.0034 (10)	−0.0035 (10)	0.0043 (10)
C3	0.0539 (13)	0.0559 (14)	0.0472 (13)	0.0003 (11)	−0.0010 (10)	0.0044 (11)
C4	0.0569 (15)	0.0557 (16)	0.096 (2)	0.0003 (12)	−0.0026 (14)	−0.0078 (14)
C5	0.089 (2)	0.0500 (16)	0.099 (2)	0.0038 (15)	0.0033 (17)	−0.0065 (14)
C6	0.099 (2)	0.0605 (17)	0.0601 (16)	0.0193 (16)	0.0048 (15)	0.0053 (13)
C7	0.084 (2)	0.088 (2)	0.0758 (19)	0.0364 (18)	−0.0124 (16)	0.0035 (16)
C8	0.0663 (17)	0.0726 (18)	0.0681 (17)	0.0130 (13)	−0.0174 (13)	−0.0053 (13)
C9	0.0360 (10)	0.0371 (11)	0.0462 (12)	−0.0018 (8)	−0.0006 (9)	−0.0015 (9)
C10	0.0427 (12)	0.0605 (14)	0.0470 (13)	−0.0016 (10)	−0.0025 (10)	−0.0043 (10)
C11	0.0347 (12)	0.0830 (17)	0.0642 (16)	0.0008 (11)	−0.0079 (11)	−0.0086 (13)
C12	0.0335 (12)	0.0918 (19)	0.0674 (17)	−0.0053 (12)	0.0092 (11)	−0.0139 (14)
C13	0.0476 (13)	0.0662 (15)	0.0500 (13)	−0.0069 (11)	0.0083 (11)	−0.0080 (11)
C14	0.0360 (11)	0.0476 (12)	0.0435 (12)	−0.0046 (9)	0.0004 (9)	−0.0022 (9)
C15	0.0489 (12)	0.0590 (14)	0.0394 (12)	−0.0133 (11)	0.0069 (10)	−0.0066 (10)
C16	0.0681 (16)	0.0558 (15)	0.0622 (15)	−0.0101 (12)	0.0007 (12)	−0.0043 (12)
C17	0.113 (3)	0.0540 (17)	0.086 (2)	−0.0182 (16)	0.0076 (19)	−0.0043 (14)
C18	0.092 (2)	0.083 (2)	0.088 (2)	−0.0443 (18)	0.0201 (18)	−0.0323 (18)
C19	0.0768 (19)	0.097 (2)	0.0630 (17)	−0.0370 (17)	0.0086 (14)	−0.0254 (16)
C20	0.0581 (14)	0.0802 (17)	0.0471 (13)	−0.0148 (13)	0.0009 (11)	−0.0107 (12)
C21	0.0491 (13)	0.0673 (15)	0.0473 (13)	−0.0062 (12)	0.0043 (10)	0.0060 (12)
C22	0.0755 (18)	0.091 (2)	0.0469 (15)	−0.0106 (15)	0.0085 (13)	−0.0012 (13)
C23	0.0714 (18)	0.123 (3)	0.0512 (16)	−0.0050 (18)	0.0115 (13)	0.0022 (17)
C24	0.114 (3)	0.154 (4)	0.067 (2)	0.022 (3)	0.0348 (19)	0.042 (2)
C25	0.161 (4)	0.100 (3)	0.105 (3)	0.024 (3)	0.047 (3)	0.047 (2)
C26	0.150 (3)	0.088 (2)	0.090 (2)	0.019 (2)	0.061 (2)	0.0298 (18)
C27	0.0638 (15)	0.0481 (13)	0.0483 (13)	0.0064 (11)	0.0064 (11)	0.0051 (10)
C28	0.0780 (18)	0.0604 (16)	0.0767 (18)	−0.0021 (14)	0.0118 (14)	−0.0049 (14)
C29	0.111 (3)	0.0569 (18)	0.090 (2)	−0.0035 (18)	0.029 (2)	−0.0093 (16)
C30	0.145 (3)	0.069 (2)	0.076 (2)	0.039 (2)	0.041 (2)	0.0141 (17)
C31	0.138 (3)	0.115 (3)	0.084 (2)	0.073 (3)	−0.012 (2)	0.006 (2)
C32	0.104 (2)	0.0723 (18)	0.0711 (18)	0.0271 (17)	−0.0163 (17)	−0.0026 (14)
C33	0.0479 (12)	0.0562 (14)	0.0450 (12)	0.0088 (11)	−0.0031 (10)	−0.0020 (10)
C34	0.0757 (18)	0.0842 (19)	0.0491 (15)	0.0040 (14)	−0.0061 (13)	0.0033 (13)
C35	0.094 (2)	0.111 (3)	0.0451 (15)	0.014 (2)	−0.0128 (14)	−0.0116 (16)
C36	0.092 (2)	0.097 (2)	0.075 (2)	0.0012 (19)	−0.0113 (17)	−0.0382 (18)
C37	0.091 (2)	0.0646 (17)	0.077 (2)	−0.0046 (15)	0.0014 (16)	−0.0201 (15)
C38	0.0625 (15)	0.0575 (15)	0.0546 (14)	0.0037 (12)	0.0040 (11)	−0.0075 (12)
C39	0.0560 (14)	0.0681 (16)	0.0477 (13)	−0.0055 (12)	0.0069 (11)	0.0069 (11)
C40	0.0454 (13)	0.0728 (16)	0.0492 (13)	−0.0041 (11)	0.0100 (10)	−0.0038 (11)
C41	0.0444 (12)	0.0579 (14)	0.0522 (13)	0.0032 (11)	0.0103 (10)	−0.0022 (11)
C42	0.0787 (18)	0.0628 (17)	0.0718 (18)	−0.0018 (14)	−0.0072 (14)	−0.0117 (13)
C43	0.115 (3)	0.0577 (17)	0.082 (2)	0.0130 (17)	0.0013 (18)	−0.0129 (14)
C44	0.091 (2)	0.083 (2)	0.108 (3)	0.0334 (19)	0.018 (2)	0.0008 (19)
C45	0.0572 (18)	0.085 (2)	0.141 (3)	0.0117 (16)	0.0014 (18)	0.010 (2)
C46	0.0510 (15)	0.0648 (17)	0.104 (2)	0.0061 (13)	−0.0028 (14)	−0.0014 (15)
C47	0.0409 (12)	0.0489 (12)	0.0554 (13)	−0.0004 (10)	0.0021 (10)	−0.0018 (10)
C48	0.0484 (14)	0.0686 (17)	0.0848 (18)	−0.0083 (12)	0.0186 (13)	−0.0168 (14)
C49	0.0475 (14)	0.0739 (18)	0.099 (2)	−0.0160 (13)	0.0126 (14)	0.0011 (16)
C50	0.0646 (16)	0.0532 (14)	0.0797 (18)	−0.0089 (13)	−0.0098 (14)	−0.0012 (13)
C51	0.0692 (17)	0.0595 (16)	0.0702 (17)	−0.0003 (13)	0.0050 (14)	−0.0128 (13)
C52	0.0503 (13)	0.0614 (15)	0.0548 (14)	−0.0054 (11)	0.0053 (11)	−0.0049 (11)
C53	0.095 (2)	0.085 (2)	0.124 (3)	−0.002 (2)	0.023 (2)	0.008 (2)
C54A	0.122 (9)	0.263 (15)	0.195 (13)	−0.032 (9)	−0.013 (8)	0.081 (13)
Cl1A	0.159 (3)	0.172 (3)	0.267 (4)	−0.043 (2)	−0.016 (3)	0.060 (3)
Cl2A	0.207 (4)	0.462 (9)	0.195 (4)	0.047 (6)	0.012 (3)	0.067 (5)
C55	0.230 (9)	0.196 (8)	0.416 (15)	0.034 (6)	0.002 (9)	−0.029 (8)
C56	0.175 (6)	0.245 (8)	0.241 (8)	−0.032 (6)	−0.006 (5)	−0.001 (6)
C57	0.217 (10)	0.264 (11)	0.179 (6)	−0.084 (8)	−0.020 (6)	0.001 (8)

### Geometric parameters (Å, °)

**Table:**

Pd1—C9	2.0783 (19)	C27—C28	1.384 (3)
Pd1—C1	2.1711 (19)	C28—C29	1.378 (4)
Pd1—P2	2.2783 (6)	C28—H28	0.9300
Pd1—P3	2.3554 (6)	C29—C30	1.351 (4)
S1—O4	1.413 (2)	C29—H29	0.9300
S1—O5	1.414 (2)	C30—C31	1.348 (5)
S1—O6	1.466 (3)	C30—H30	0.9300
S1—C53	1.777 (4)	C31—C32	1.389 (4)
P1—C1	1.768 (2)	C31—H31	0.9300
P1—C14	1.783 (2)	C32—H32	0.9300
P1—C21	1.797 (2)	C33—C38	1.379 (3)
P1—C15	1.800 (2)	C33—C34	1.402 (3)
P3—C41	1.818 (2)	C34—C35	1.367 (4)
P3—C47	1.819 (2)	C34—H34	0.9300
P3—C40	1.834 (2)	C35—C36	1.356 (4)
P2—C33	1.814 (2)	C35—H35	0.9300
P2—C27	1.817 (2)	C36—C37	1.352 (4)
P2—C39	1.834 (2)	C36—H36	0.9300
O1—C2	1.218 (2)	C37—C38	1.378 (3)
O2—N1	1.182 (4)	C37—H37	0.9300
O3—N1	1.159 (4)	C38—H38	0.9300
F1—C53	1.346 (4)	C39—C40	1.517 (3)
F2—C53	1.332 (4)	C39—H39A	0.9700
F3—C53	1.324 (4)	C39—H39B	0.9700
N1—C6	1.512 (4)	C40—H40A	0.9700
C1—C2	1.471 (3)	C40—H40B	0.9700
C1—H1	0.9800	C41—C46	1.378 (3)
C2—C3	1.504 (3)	C41—C42	1.381 (3)
C3—C4	1.383 (3)	C42—C43	1.397 (4)
C3—C8	1.397 (3)	C42—H42	0.9300
C4—C5	1.392 (3)	C43—C44	1.358 (4)
C4—H4	0.9300	C43—H43	0.9300
C5—C6	1.334 (4)	C44—C45	1.376 (4)
C5—H5	0.9300	C44—H44	0.9300
C6—C7	1.361 (4)	C45—C46	1.377 (4)
C7—C8	1.375 (4)	C45—H45	0.9300
C7—H7	0.9300	C46—H46	0.9300
C8—H8	0.9300	C47—C52	1.381 (3)
C9—C10	1.397 (3)	C47—C48	1.390 (3)
C9—C14	1.405 (3)	C48—C49	1.368 (3)
C10—C11	1.378 (3)	C48—H48	0.9300
C10—H10	0.9300	C49—C50	1.383 (4)
C11—C12	1.363 (3)	C49—H49	0.9300
C11—H11	0.9300	C50—C51	1.352 (3)
C12—C13	1.380 (3)	C50—H50	0.9300
C12—H12	0.9300	C51—C52	1.378 (3)
C13—C14	1.378 (3)	C51—H51	0.9300
C13—H13	0.9300	C52—H52	0.9300
C15—C16	1.379 (3)	C54A—Cl1A	1.726 (8)
C15—C20	1.389 (3)	C54A—Cl2A	1.730 (8)
C16—C17	1.394 (3)	C54A—H54A	0.9700
C16—H16	0.9300	C54A—H54B	0.9700
C17—C18	1.370 (4)	C54B—Cl2B	1.746 (9)
C17—H17	0.9300	C54B—Cl1B	1.787 (9)
C18—C19	1.361 (4)	C54B—H54C	0.9700
C18—H18	0.9300	C54B—H54D	0.9700
C19—C20	1.381 (3)	C54C—Cl2C	1.744 (10)
C19—H19	0.9300	C54C—Cl1C	1.778 (10)
C20—H20	0.9300	C54C—H54E	0.9700
C21—C26	1.352 (4)	C54C—H54F	0.9700
C21—C22	1.386 (3)	C55—C56	1.472 (6)
C22—C23	1.384 (4)	C55—H55A	0.9600
C22—H22	0.9300	C55—H55B	0.9600
C23—C24	1.310 (4)	C55—H55C	0.9600
C23—H23	0.9300	C56—C57	1.523 (7)
C24—C25	1.357 (5)	C56—H56A	0.9700
C24—H24	0.9300	C56—H56B	0.9700
C25—C26	1.401 (4)	C57—C57^i^	1.455 (8)
C25—H25	0.9300	C57—H57A	0.9700
C26—H26	0.9300	C57—H57B	0.9700
C27—C32	1.375 (3)		
			
C9—Pd1—C1	83.17 (8)	C30—C29—C28	120.0 (3)
C9—Pd1—P2	92.50 (6)	C30—C29—H29	120.0
C1—Pd1—P2	175.31 (5)	C28—C29—H29	120.0
C9—Pd1—P3	175.68 (5)	C31—C30—C29	120.6 (3)
C1—Pd1—P3	100.49 (5)	C31—C30—H30	119.7
P2—Pd1—P3	83.91 (2)	C29—C30—H30	119.7
O4—S1—O5	116.71 (16)	C30—C31—C32	120.7 (3)
O4—S1—O6	112.71 (18)	C30—C31—H31	119.7
O5—S1—O6	114.10 (17)	C32—C31—H31	119.7
O4—S1—C53	104.18 (18)	C27—C32—C31	119.5 (3)
O5—S1—C53	104.67 (17)	C27—C32—H32	120.3
O6—S1—C53	102.43 (19)	C31—C32—H32	120.3
C1—P1—C14	100.26 (9)	C38—C33—C34	118.5 (2)
C1—P1—C21	114.63 (11)	C38—C33—P2	120.54 (17)
C14—P1—C21	110.91 (10)	C34—C33—P2	120.85 (19)
C1—P1—C15	116.56 (10)	C35—C34—C33	119.5 (3)
C14—P1—C15	108.63 (10)	C35—C34—H34	120.2
C21—P1—C15	105.72 (10)	C33—C34—H34	120.2
C41—P3—C47	106.47 (10)	C36—C35—C34	120.7 (3)
C41—P3—C40	103.43 (10)	C36—C35—H35	119.6
C47—P3—C40	101.05 (10)	C34—C35—H35	119.6
C41—P3—Pd1	123.64 (7)	C37—C36—C35	120.8 (3)
C47—P3—Pd1	114.05 (7)	C37—C36—H36	119.6
C40—P3—Pd1	105.28 (7)	C35—C36—H36	119.6
C33—P2—C27	108.75 (10)	C36—C37—C38	119.9 (3)
C33—P2—C39	102.42 (11)	C36—C37—H37	120.0
C27—P2—C39	104.28 (11)	C38—C37—H37	120.0
C33—P2—Pd1	113.60 (7)	C37—C38—C33	120.5 (2)
C27—P2—Pd1	117.51 (7)	C37—C38—H38	119.8
C39—P2—Pd1	108.79 (8)	C33—C38—H38	119.8
O3—N1—O2	126.6 (4)	C40—C39—P2	107.48 (15)
O3—N1—C6	117.0 (4)	C40—C39—H39A	110.2
O2—N1—C6	116.4 (3)	P2—C39—H39A	110.2
C2—C1—P1	118.04 (15)	C40—C39—H39B	110.2
C2—C1—Pd1	119.69 (14)	P2—C39—H39B	110.2
P1—C1—Pd1	99.62 (9)	H39A—C39—H39B	108.5
C2—C1—H1	106.1	C39—C40—P3	107.53 (15)
P1—C1—H1	106.1	C39—C40—H40A	110.2
Pd1—C1—H1	106.1	P3—C40—H40A	110.2
O1—C2—C1	122.1 (2)	C39—C40—H40B	110.2
O1—C2—C3	119.31 (19)	P3—C40—H40B	110.2
C1—C2—C3	118.59 (19)	H40A—C40—H40B	108.5
C4—C3—C8	118.1 (2)	C46—C41—C42	118.7 (2)
C4—C3—C2	124.1 (2)	C46—C41—P3	123.39 (19)
C8—C3—C2	117.8 (2)	C42—C41—P3	117.94 (18)
C3—C4—C5	120.6 (2)	C41—C42—C43	120.0 (3)
C3—C4—H4	119.7	C41—C42—H42	120.0
C5—C4—H4	119.7	C43—C42—H42	120.0
C6—C5—C4	119.0 (3)	C44—C43—C42	120.0 (3)
C6—C5—H5	120.5	C44—C43—H43	120.0
C4—C5—H5	120.5	C42—C43—H43	120.0
C5—C6—C7	122.8 (3)	C43—C44—C45	120.7 (3)
C5—C6—N1	118.5 (3)	C43—C44—H44	119.6
C7—C6—N1	118.7 (3)	C45—C44—H44	119.6
C6—C7—C8	119.0 (3)	C44—C45—C46	119.1 (3)
C6—C7—H7	120.5	C44—C45—H45	120.4
C8—C7—H7	120.5	C46—C45—H45	120.4
C7—C8—C3	120.5 (3)	C45—C46—C41	121.5 (3)
C7—C8—H8	119.7	C45—C46—H46	119.3
C3—C8—H8	119.7	C41—C46—H46	119.3
C10—C9—C14	115.00 (18)	C52—C47—C48	117.5 (2)
C10—C9—Pd1	128.35 (16)	C52—C47—P3	120.37 (16)
C14—C9—Pd1	116.65 (14)	C48—C47—P3	121.90 (17)
C11—C10—C9	121.6 (2)	C49—C48—C47	121.1 (2)
C11—C10—H10	119.2	C49—C48—H48	119.4
C9—C10—H10	119.2	C47—C48—H48	119.4
C12—C11—C10	121.6 (2)	C48—C49—C50	120.5 (2)
C12—C11—H11	119.2	C48—C49—H49	119.8
C10—C11—H11	119.2	C50—C49—H49	119.8
C11—C12—C13	119.3 (2)	C51—C50—C49	118.7 (2)
C11—C12—H12	120.4	C51—C50—H50	120.7
C13—C12—H12	120.4	C49—C50—H50	120.7
C14—C13—C12	119.0 (2)	C50—C51—C52	121.5 (2)
C14—C13—H13	120.5	C50—C51—H51	119.2
C12—C13—H13	120.5	C52—C51—H51	119.2
C13—C14—C9	123.56 (19)	C51—C52—C47	120.6 (2)
C13—C14—P1	124.16 (16)	C51—C52—H52	119.7
C9—C14—P1	112.28 (14)	C47—C52—H52	119.7
C16—C15—C20	119.9 (2)	F3—C53—F2	105.1 (3)
C16—C15—P1	120.01 (17)	F3—C53—F1	109.0 (3)
C20—C15—P1	120.12 (19)	F2—C53—F1	109.5 (3)
C15—C16—C17	119.1 (2)	F3—C53—S1	112.8 (3)
C15—C16—H16	120.4	F2—C53—S1	110.9 (3)
C17—C16—H16	120.4	F1—C53—S1	109.4 (3)
C18—C17—C16	120.3 (3)	Cl1A—C54A—Cl2A	106.1 (6)
C18—C17—H17	119.8	Cl1A—C54A—H54A	110.5
C16—C17—H17	119.8	Cl2A—C54A—H54A	110.5
C19—C18—C17	120.6 (3)	Cl1A—C54A—H54B	110.5
C19—C18—H18	119.7	Cl2A—C54A—H54B	110.5
C17—C18—H18	119.7	H54A—C54A—H54B	108.7
C18—C19—C20	120.0 (3)	Cl2B—C54B—Cl1B	106.2 (8)
C18—C19—H19	120.0	Cl2B—C54B—H54C	110.5
C20—C19—H19	120.0	Cl1B—C54B—H54C	110.5
C19—C20—C15	120.1 (3)	Cl2B—C54B—H54D	110.5
C19—C20—H20	120.0	Cl1B—C54B—H54D	110.5
C15—C20—H20	120.0	H54C—C54B—H54D	108.7
C26—C21—C22	118.4 (2)	Cl2C—C54C—Cl1C	96.1 (7)
C26—C21—P1	120.5 (2)	Cl2C—C54C—H54E	112.5
C22—C21—P1	121.1 (2)	Cl1C—C54C—H54E	112.5
C23—C22—C21	120.6 (3)	Cl2C—C54C—H54F	112.5
C23—C22—H22	119.7	Cl1C—C54C—H54F	112.5
C21—C22—H22	119.7	H54E—C54C—H54F	110.1
C24—C23—C22	120.6 (3)	C56—C55—H55A	109.5
C24—C23—H23	119.7	C56—C55—H55B	109.5
C22—C23—H23	119.7	H55A—C55—H55B	109.5
C23—C24—C25	120.1 (3)	C56—C55—H55C	109.5
C23—C24—H24	119.9	H55A—C55—H55C	109.5
C25—C24—H24	119.9	H55B—C55—H55C	109.5
C24—C25—C26	120.6 (3)	C55—C56—C57	112.2 (6)
C24—C25—H25	119.7	C55—C56—H56A	109.2
C26—C25—H25	119.7	C57—C56—H56A	109.2
C21—C26—C25	119.3 (3)	C55—C56—H56B	109.2
C21—C26—H26	120.3	C57—C56—H56B	109.2
C25—C26—H26	120.3	H56A—C56—H56B	107.9
C32—C27—C28	118.8 (2)	C57^i^—C57—C56	123.4 (9)
C32—C27—P2	123.8 (2)	C57^i^—C57—H57A	106.5
C28—C27—P2	117.34 (19)	C56—C57—H57A	106.5
C29—C28—C27	120.4 (3)	C57^i^—C57—H57B	106.5
C29—C28—H28	119.8	C56—C57—H57B	106.5
C27—C28—H28	119.8	H57A—C57—H57B	106.5

Symmetry codes: (i) −*x*, −*y*+1, −*z*.

### Hydrogen-bond geometry (Å, °)

**Table:**

*D*—H···*A*	*D*—H	H···*A*	*D*···*A*	*D*—H···*A*
C19—H19···O1^ii^	0.93	2.54	3.284 (3)	138
C30—H30···O6^iii^	0.93	2.55	3.372 (4)	148
C39—H39B···O6^iv^	0.97	2.59	3.309 (4)	131

Symmetry codes: (ii) −*x*+1, −*y*, −*z*; (iii) *x*, *y*−1, *z*; (iv) −*x*+3/2, *y*−1/2, −*z*+1/2.

## References

[bb1] Aguilar, D., Aragues, M. A., Bielsa, R., Serrano, E., Soler, T., Navarro, R. & Urriolabeitia, E. P. (2008). *J. Organomet. Chem.* **693**, 417–424.

[bb2] Altomare, A., Burla, M. C., Camalli, M., Cascarano, G. L., Giacovazzo, C., Guagliardi, A., Moliterni, A. G. G., Polidori, G. & Spagna, R. (1999). *J. Appl. Cryst.* **32**, 115–119.

[bb3] Bruker (2008). *APEX2*, *SAINT* and *SADABS* Bruker AXS Inc., Madison, Wisconsin, USA.

[bb4] Chen, X., Engle, K. M., Wang, D.-H. & Yu, J.-Q. (2009). *Angew. Chem. Int. Ed.* **48**, 5094–5115. 10.1002/anie.200806273 PMC2722958 19557755

[bb5] Dupont, J., Pfeffer, M. & Spencer, J. (2001). *Eur. J. Inorg. Chem.* pp. 1917–1927.

[bb6] Falvello, L. R., Fernandez, S., Navarro, R., Rueda, A. & Urriolabeitia, E. P. (1998). *Organometallics*, **17**, 5887–5900.

[bb7] Falvello, L. R., Fernandez, S., Navarro, R. & Urriolabeitia, E. P. (1999). *Inorg. Chem.* **38**, 2455–2463. 10.1021/ic990923z 11232839

[bb8] Farrugia, L. J. (1997). *J. Appl. Cryst.* **30**, 565.

[bb9] Karami, K., Büyükgüngör, O. & Dalvand, H. (2010). *Transition Met. Chem.* **35**, 621–626.

[bb10] Karami, K., Rizzoli, C. & Borzooie, F. (2011). *Polyhedron*, **30**, 778–784.

[bb11] Karami, K., Rizzoli, C. & Salah, M. M. (2011). *J. Organomet. Chem.* **696**, 940–945.

[bb12] Keller, E. (1997). *SCHAKAL97* University of Freiburg, Germany.

[bb13] Nardelli, M. (1995). *J. Appl. Cryst.* **28**, 659.

[bb14] Rietling, V., Sirlin, C. & Pfeffer, M. (2002). *Chem. Rev.* **102**, 1731–1770. 10.1021/cr0104330 11996548

[bb15] Shao, M., Jin, X., Tang, Y., Huang, Q. & Huang, Y. (1982). *Tetrahedron Lett.* **23**, 5343–5346.

[bb16] Sheldrick, G. M. (2008). *Acta Cryst.* A**64**, 112–122. 10.1107/S0108767307043930 18156677

